# UHPLC-MS Metabolomic Fingerprinting, Antioxidant, and Enzyme Inhibition Activities of *Himantormia lugubris* from Antarctica

**DOI:** 10.3390/metabo12060560

**Published:** 2022-06-18

**Authors:** Carlos Areche, Javier Romero Parra, Beatriz Sepulveda, Olimpo García-Beltrán, Mario J. Simirgiotis

**Affiliations:** 1 Departamento de Química, Facultad de Ciencias, Universidad de Chile, Las Palmeras 3425, Nuñoa, Santiago 7800024, Chile; 2 Departamento de Química Orgánica y Fisicoquímica, Facultad de Ciencias Químicas y Farmacéuticas, Universidad de Chile, Olivos 1007, Casilla, Santiago 6640022, Chile; javier.romero@ciq.uchile.cl; 3 Departamento de Ciencias Químicas, Viña del Mar, Universidad Andres Bello, Viña del Mar 2520000, Chile; bsepulveda@uc.cl; 4 Facultad de Ciencias Naturales y Matemáticas, Universidad de Ibagué, Carrera 22 Calle 67, Ibagué 730001, Colombia; jose.garcia@unibague.edu.co; 5 Instituto de Farmacia, Facultad de Ciencias, Universidad Austral de Chile, Elena Haverbeck S-N, Valdivia 5090000, Chile

**Keywords:** *Himantormia*, phenolics, enzyme inhibition, native lichens, antioxidant, depsides, dibenzofurans, Antarctica, Alzheimer

## Abstract

*Himantormia lugubris* is a Chilean native small lichen shrub growing in the Antarctica region. In this study, the metabolite fingerprinting and the antioxidant and enzyme inhibitory potential from this species and its four major isolated compounds were investigated for the first time. Using ultra-high performance liquid chromatography coupled to quadrupole-Orbitrap mass spectrometry analysis (UHPLC-Q-Orbitrap-MS), several metabolites were identified including specific compounds as chemotaxonomical markers, while major metabolites were quantified in this species. A good inhibition activity against cholinesterase (acetylcholinesterase (AChE) IC_50_: 12.38 ± 0.09 µg/mL, butyrylcholinesterase (BChE) IC_50_: 31.54 ± 0.20 µg/mL) and tyrosinase (22.32 ± 0.21 µg/mL) enzymes of the alcoholic extract and the main compounds (IC_50_: 28.82 ± 0.10 µg/mL, 36.43 ± 0.08 µg/mL, and 7.25 ± 0.18 µg/mL, respectively, for the most active phenolic atranol) was found. The extract showed a total phenolic content of 47.4 + 0.0 mg of gallic acid equivalents/g. In addition, antioxidant activity was assessed using bleaching of DPPH and ORAC (IC_50_: 75.3 ± 0.02 µg/mL and 32.7 ± 0.7 μmol Trolox/g lichen, respectively) and FRAP (27.8 ± 0.0 μmol Trolox equivalent/g) experiments. The findings suggest that *H. lugubris* is a rich source of bioactive compounds with potentiality in the prevention of neurodegenerative or noncommunicable chronic diseases.

## 1. Introduction

Plant-like organisms formed by the symbiotic association of cyanobacteria or algae and fungi are known as lichens. They are known as an organism’s community rather than a simple fungi–algae association. There are around 15,000 lichen species found worldwide and they occur in several different environmental conditions such as in dry deserts or Antarctica and usually grow freely on trees, rocks, or in soil. They are used as dye or food and provide support to animals such as reindeers and have medicinal properties supported by the presence of phenolic compounds. In recent years, some phenolic compounds isolated from lichens proved to be active in the prevention of noncommunicable chronic diseases [1] and related ailments such as Alzheimer’s disease and cancer. For instance, usnic acid, a main phenolic compound in the majority of lichens, showed activity against the p53 MCF7 and MDA-MB-231 breast cancer cell lines plus the H1299 lung cancer cell line [2,3]. It also showed anti-inflammatory [4], antifungal, and antimicrobial activities [5,6], among others. The mechanism of action for biological activities of some of the phenolics from lichens is usually related to their antioxidant capacity [7,8] as in the case of the lichenic compound fumarprotocetraric acid, which attenuated intracellular reactive oxygen species (ROS) formation, lipid peroxidation, and glutathione (GSH) depletion [9]. Some phenolics from lichens were effective as inhibitors of enzymes implicated in chronic diseases. For instance, usnic acid, salazinic acid, and sekikaic acid were effective against the glucosidase enzyme, which is involved in the metabolic syndrome, with the highest activity shown by sekikaic acid [10], among other important bioactivities.

**Figure 1:**
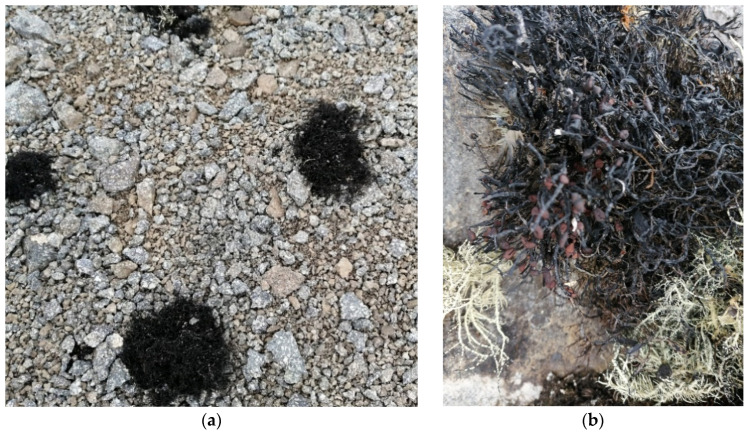
(**a**) *H. lugubris* growing on King George Island collected in February 2021. (**b**) Close picture.

*Himantormia lugubris* is an Antarctic endemic lichen distributed in the Antarctic Peninsula, King George, South Georgia, Ardley, and adjacent islands and is an important habitant of the community of epilithic lichens [11]. The species has flattened branches with a thick thallus, while its grey surface is often disrupted, revealing the black and superior chondroid axis (Figure 1).

The inhibition of cholinesterase enzymes plays a role in the therapy of Alzheimer’s disease. Acetylcholinesterase (AChE) and butyrylcholinesterase (BChE) are related enzymes found in animals. AChE has a critical role in neurotransmission, and a physiological role for BChE is just now emerging. Some irreversible cholinesterase inhibitors are used conversely for their toxic prospective as chemical weapons and pesticides, but some reversible and competitive nootropic cholinesterase inhibitor alkaloids such as galantamine are today used for the treatment of Alzheimer’s disease. Several isolated lichenic compounds have been examined for anti-acetylcholinesterase use and may play a fundamental role in the prevention of this dementia. For instance, lichesterinic acid isolated previously from a sample of *H. lugubris* inhibited the progression of tau aggregates involved in Alzheimer’s disease, postulated to be through the Michael reaction of the unsaturated carbonyl moieties of the lichen compounds and cysteine moieties of the aggregates [12], while usnic acid, the main active dibenzofuran derivative from lichens, and some of its derivatives showed good anti-cholinergic activity [13]. In addition, some lichenic compounds have proved to be tyrosinase inhibitors [14]. The enzyme tyrosinase is responsible for the browning color in humans, microorganisms, and plants; it is a multifunctional copper-containing enzyme and a key factor in the melanin synthesis widely explored as a target of modulatory agents of melanization. Melanin in human skin is a crucial pigment for protection against UV-induced damage, but excessive melanin can provoke pigmentation disorders, such as ephelides, melasma, and lentigines [14]. Neuromelanin, which is a related melanin in the dopamine neurons is decreased in Parkinson’s disease, the second most common neurodegenerative disease after Alzheimer’s disease [15]. Some phenolic resorcinol derivatives were proposed as tyrosinase inhibitors as a novel strategy for the treatment of Parkinson’s disease, to improve locomotor capacity [16]. Furthermore, some orsellinic acid derivatives, the main phenolics isolates from the lichen *Parmotrema*, were also shown to be highly active tyrosinase inhibitors [17]. However, there are no scientific reports concerning the enzyme inhibition potential of those important enzymes by extracts or other compounds present in *H. lugubris* from Antarctica, which could be interesting for the treatment or prevention of those chronic neurodegenerative diseases.

Ultra-high-resolution chromatography (UHPLC) coupled to mass spectrometry (MS) is a rapid and modern tool providing exact information and comparison of the chemical profile of different metabolites present in shrubs and fruits [18,19]. Our group has investigated the metabolome fingerprinting of Chilean native lichens and plants using this technique, and the antioxidant and enzyme inhibitory properties were also explored [20,21,22,23,24]. In this work, we report the metabolome fingerprinting of *H. lugubris* ethanolic extract by UHPLC-MS analysis, plus its antioxidant activity and enzyme inhibitory potential (against cholinesterase and tyrosinase), with full docking experiments of the extract and four isolated compounds for the first time. 

## 2. Results and Discussion

### 2.1. UHPLC Chromatographic Analysis of Himantormia lugubris Extracts

**Figure 2:**
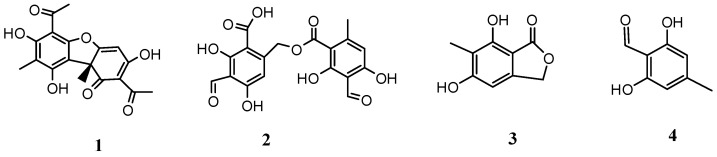
Compounds isolated from *H. lugubris* (Usnic acid, **1**; barbatolic acid, **2**; 5,7-dihydroxy-6-methylphthalide, **3**; and atranol, **4**).

**Figure 3:**
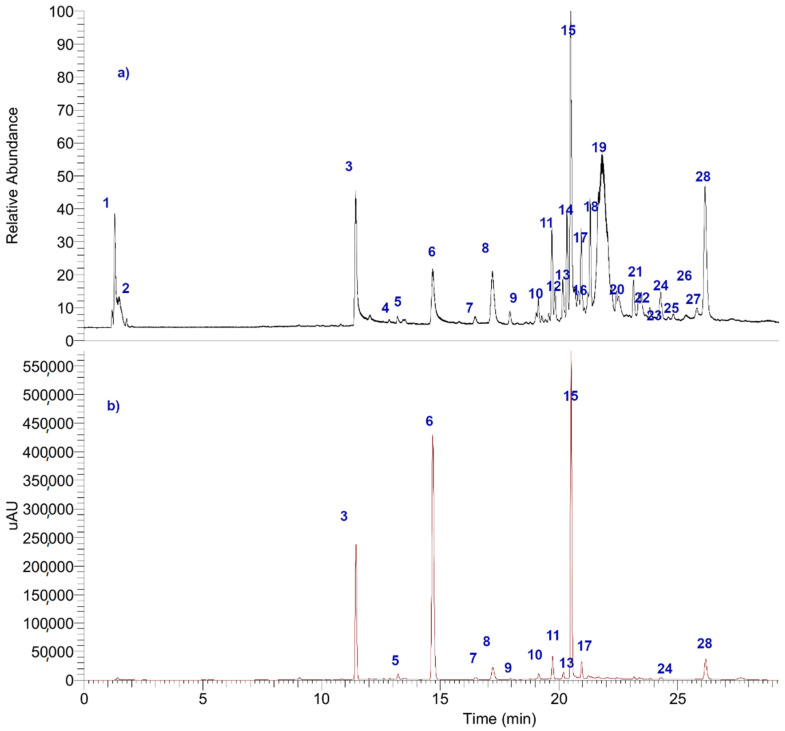
(**a**) UHPLC-TIC chromatogram and (**b**) UHPLC-UV chromatogram at 280 nm of *H. lugubris* ethanolic extract. The peak numbers correspond to those identified in Table 1.

Four compounds were isolated from the ethanolic extract of *H. lugubris* (Figure 2); in addition, the fingerprint analysis was obtained by means of high-resolution mass spectrometric analysis (UHPLC-MS) (Figure 3). Twenty-eight compounds were detected and tentatively identified, based on HR-MS fragmentation patterns (Table 1). In addition, the main four compounds were quantified in the extract by HPLC UV absorbance (Table 2, Figure 2). The metabolites identified in this species were mainly depsides, depsidones, lipids, diphenyl-ether derivatives, and dibenzofurans. The detailed fingerprinting analysis is explained below. 

#### 2.1.1. Aromatic Derivatives

Peak 4 was identified as 5,7-dihydroxy-4-methylphthalide and peak 6 as its isomer 5,7-dihydroxy-6-methylphthalide (C_9_H_7_O_4_), while peak 3 was identified as atranol (C_8_H_7_O_3_), peak 5 as haematommic acid (C_9_H_7_O_5_), and peak 16 as haematommic acid isomer (possibly the 3-formyl-2,5-dihydroxy-6-methylbenzoic acid). Peak 10 was identified as methyl orsellinate (C_9_H_9_O_4_), peak 14 as barbatolic acid [25], and peak 17 as 2,6-diformyl-3,5-dihydroxytoluene. These findings are in good agreement with the presence of those compounds in *Usnea* lichens by Salgado et al. [25].

#### 2.1.2. Carbohydrates or Derivatives

Peaks 1 and 2 were identified as mannitol and citric acid (C_6_H_13_O_6_ and C_6_H_7_O_7_), respectively.

#### 2.1.3. Fatty Acids

Peaks 7 and 8 were identified as the dietary important oxylipin lipids: 9,10,12,13-tetrahydroxyheneicosanoic acid and 9,10,12,13,14-pentahydroxytetracosanoic acid (C_21_H_41_O_6,_ C_24_H_47_O_7_), respectively, while peak 11 was identified with an [M-H]^−^ ion at *m*/*z* 403.3047 as the related 9,10,12,13-tetrahydroxydocosanoic acid (C_22_H_43_O_6_). Peak 13 was identified as pentahydroxyhexacosanoic acid (C_26_H_51_O_7_), peak 15 as 9,10,12,13-tetrahydroxytricosanoic acid (C_23_H_45_O_6_), peak 19 as 9,10,12,13,14,15-hexahydroxyheptacosenoic acid, peak 20 as methyl 9,10,11,12,13-pentahydroxy-14-oxoheptacosanoate (C_28_H_53_O_8_), peaks 22 and 23 as tetrahydroxydioxoheneicosanoic acid and tetrahydroxydocosanoic acid, respectively, and peak 21 as lichesterinic acid (C_19_H_31_O_4_). The presence of unsaturated fatty acids in lichens was previously reported by us [24].

#### 2.1.4. Depsides

Peak 18 with a molecular anion at *m*/*z* 331.0818 and diagnostic peaks at *m*/*z* 167.0334; 151.0386; 135.0437; 313.0703; 123.0439; and 181.0494 was identified as evernic acid (2-hydroxy-4-(2-hydroxy-4-methoxy-6-methylbenzoyl)oxy-6-methylbenzoic acid, C_17_H_15_O_7_), while peak 9 was identified as its isomer (3-hydroxy-4-(2-hydroxy-4-methoxy-6-methylbenzoyl)oxy-6-methylbenzoic acid) and peak 12 as another isomer, possibly the 3-hydroxy-4-(3-hydroxy-4-methoxy-6-methylbenzoyl)oxy-6-methylbenzoic acid, and peak 24 was tentatively identified as sphaerophorin (C_23_H_27_O_7_). Peak 27 with a molecular anion at *m*/*z* 359.1120 and daughter ions at *m*/*z* 181.0493; 163.0387; and 137.0594 was identified as barbatic acid (2-hydroxy-4-(2-hydroxy-4-methoxy-3,6-dimethylbenzoyl) oxy-3,6-dimethylbenzoic acid, C_19_H_19_O_7_), and peak 26 was identified as its isomer, possibly 3-hydroxy-4-(2-hydroxy-4-methoxy-3,6-dimethylbenzoyl) oxy-3,6-dimethylbenzoic acid. Most of the described depsides were previously reported in lichens by Torres-Benitez et al. [24].

#### 2.1.5. Dibenzofurans

Peak 28 was identified as usnic acid (C_18_H_15_O_7_) and peak 25 as pseudoplacodiolic acid or placodiolic acid (C_19_H_19_O_8_). Those compounds were previously reported in *Usnea* lichens by Salgado et al. [25].

### 2.2. Quantitative Analysis

Usnic acid has been reported as the most important bioactive compound in several lichens, and mostly extracts of the genus Usnea [26] because of its abundance, easy isolation, multiple biological activities, and promising contribution as a component of phytopharmaceuticals [27,28]. The content of usnic acid in our Antarctic sample of *H. lugubris* was close to 1% measured by HPLC (Table 2), close to that reported in the lichens *Parmelia conspersa* (1.1 ± 0.09%), *Hypogymnia tubulosa* (1.24 ± 0.09%), and *Lavoparmelia caperata* (1.29 ± 0.03%) [29] and lower than that of *Usnea lichen* (1.69 ± 1.9%) [30]. Moreover, the usnic acid contents reported in several *Rhizoplaca* species were 0.19–4.0% dry weight, almost double our values [31]. Popovici et al. [32] found in *U. barbata* variable concentrations of usnic acid in four extracts obtained by different solvents: ethyl acetate (376.73 mg/g), acetone (282.78 mg/g), methanol (137.60 mg/g), and ethanol (127.21 mg/g) [33]. In *U. barbata*, the usnic acid content present in methanolic extracts [34], ethyl acetate extracts [3], and pure conditions [35] was determined as being responsible for high cytotoxicity on cancer cells, highlighting its potential antitumor activity. Pathak et al. [36] found efficient antidermatophytic activity in *U. orientalis* extract, mediated by the high concentration of usnic acid. Moreover, usnic acid in *U. longissima* is found in high concentrations and is evidenced with a significant effect on different cancer cell lines [37]. As for other chemical compounds, atranol was reported as a biologically active constituent of several lichens [38] including *Stereocaulon azoreum* and *Stereocaulon vesubianum* [39,40], and in Antarctic lichens in *Everniopsis trulla* [41], while barbatolic acid reported in *Bryoria* lichens [42] was reported to be active against breast cancer [43], showed antiangiogenic effects [44], and was previously reported in *H. lugubris* [45] while the compound 5,7-dihydroxy-6-methylphthalide was only reported to occur in *Usnea florida* [46] and *Usnea antarctica* [41]; the concentration of both compounds was 85.833 and 49.374 per gram of lichen, respectively (Table 2). 

### 2.3. Total Phenolic Contents and Antioxidant and Enzymatic Inhibitory Activity

The use of plants or lichens has been important over the years to prevent neurodegenerative diseases due to the high content of bioactive compounds [22,24,47]; some of them have been reported to have a high content of phenolics [48], and several lichens have been regarded as antioxidant [49] while specific compounds from lichens are promising therapeutic phenolics for diseases mediated by enzymes [10]. In this work, the ethanolic extract of *H. lugubris* was assessed for antioxidant activity and content of phenolic compounds (Table 2); the total phenolics were close to those published for the lichen *Usnea longissima* (38.6 mg GAE/g), and three published *Umbiricaria* (19–47 mg pyrogallol/g) [50], but lower than *Lobaria pulmonaria* (87.9 mg GAE/g) [48]. It showed moderate antioxidant activity (DPPH, IC_50_: 75.3 ± 0.02 mg/mL), while usnic acid, one of its main constituents, showed an IC_50_ of 55.25 ± 0.04 mg/mL for DPPH, which coincides with that reported previously [13], but the simpler phenolics atranol and 5,7-dihydroxy-6-methylphthalide showed more potent DPPH bleaching activity which is coincident with the other complementary tests (Table 3). The extract and pure compounds were also tested in vitro for cholinesterase and mushroom tyrosinase inhibitory potential. As far as we know, no previous reports regarding anti-enzymatic potential have been conducted for this species. The results are summarized in Table 3 and are expressed as IC_50_ values (µg/mL). Usnic acid is one of the most common and abundant lichen metabolites, and showed an IC_50_ of 1.21 and 4.36 µg/mL in AChE and BChE, which is similar to that reported previously (IC_50_: 1.27 nM and IC_50_: 3.397 nM in AChE and BChE, respectively) [13], being one of the most active compounds in the ethanolic extract, while the compound 5,7-dihydroxy-6-methylphthalide also showed good activity (12.71 ± 0.12 µg/mL and 19.47 ± 0.10 µg/mL in AChE and BChE, respectively) followed by barbatolic acid (17.42 ± 0.03, and 23.95 ± 0.02 µg/mL) and atranol (28.82 ± 0.10 and 36.43 ± 0.08 µg/mL). On the other side, the anti-tyrosinase properties of usnic acid were low (132.23 ± 0.12 μg/mL), in concordance with previous results of (+) usnic acid, where at the dose of 250 μg/mL, (+) usnic acid revealed 9.1% tyrosinase inhibition [51], while the IC_50_ value for the control kojic acid was 0.76 ± 0.05 μg/mL (Table 2). However, for the other phenolics, barbatolic acid, atranol, and 5,7-dihydroxy-6-methylphthalide (35.23 ± 0.11, 7.25 ± 0.18, and 12.13 ± 0.15 µg/mL, respectively) activity was higher; this should be due to the greater mobility and more free OH groups in the benzene rings of those compounds and the similarity of atranol’s structure with that of kojic acid, the standard inhibitor, and orsellinic acid (2,4-dihydroxy-6-methylbenzoic acid), which proved to inhibit tyrosinase activity by 63.35% and its diphenol derivative lecanoric acid by 82.69% [17]. The depsidone fumarprotocetraric acid showed inhibition at 0.6 mM of tyrosinase activity by 39.8% [52]. The activity is close to that of other phenolic compounds isolated from fruits and plants. For instance, the main phenolic constituents studied by our group showed good fitting by calculations in the catalytic site of butyryl cholinesterase enzymes [53], where polar phenolic compounds from Chilean plants studied by our research team proved to be active against these important enzymes, for instance, the glycosylated flavonoid isoastilbin [20], showing inhibitory activity which was explained by docking into catalytic sites of cholinesterase (AChE: 4.68 ± 0.03 (51.70% at 2.2 µM) and BChE: 8.51 ± 0.03 (50.10% at 2.2 µM)) enzymes. Other phenolic glycosides found by our group also showed good inhibitory activity in those enzymes, for instance, *Artemisia copa* ethanolic extract showed an IC_50_ of 3.92 ± 0.08 µg per mL in AChE and an IC_50_ of 44.13 ± 0.10 µg per mL in BChE, while some of the constituents, such as apigenin-7-*O*-glucoside and kaempferol-3-*O*-galactoside, showed binding energies of 9.76 and −2.04 kcal/mol for *Tc*AChE and −5.93 and −8.92 for *h*BChE, respectively, in the active sites of these enzymes [54]. These reports highlight the importance of phenolics and especially small phenolics in *H. lugubris* that could be suitable for use in the prevention of neurodegenerative diseases.

### 2.4. Docking Assays

All compounds shown in Figure 2, as well as the known cholinesterase inhibitor galantamine and kojic acid for tyrosine inhibition, were subjected to docking assays in the acetylcholinesterase catalytic site, butyrylcholinesterase catalytic site, and tyrosinase catalytic site to vindicate their pharmacological results and analyze their protein molecular interplay considering the experimental inhibition activities obtained (Table 3). The best docking binding energies (expressed in kcal/mol) for each compound are shown in Table 4.

#### 2.4.1. Acetylcholinesterase (TcAChE) Docking Results

Table 4 shows the binding energies of usnic acid, barbatolic acid, 7-dihydroxy-6-methylphthalide, and atranol. All the compounds displayed energy descriptors in a good manner over the acetylcholinesterase enzyme. Nonetheless, the best binding energy profile was shown by usnic acid with a value of −10.779 kcal/mol. Barbatolic acid showed the second-best energy profile but was close in magnitude to that shown by 7-dihydroxy-6-methylphthalide (−8.027 kcal/mol and 7.913 kcal/mol, respectively), which suggests that usnic acid could be mainly responsible for the acetylcholinesterase inhibitory activity. The energy descriptors obtained in our docking assays agree with the acetylcholinesterase inhibitory experiments, since galantamine possess an IC_50_ value of 0.27 + 0.03 ppm and usnic acid an IC_50_ value of 2.21 ± 0.03 ppm, finding concordance between the inhibitory potency and the energies mentioned above. In the same way, the similar energies shown by barbatolic acid and 7-dihydroxy-6-methylphthalide are also manifested in their close IC_50_ values, showing an analogous order of magnitude in their potencies (17.42 ± 0.10 ppm for barbatolic acid and 12.71 ± 0.12 ppm for 7-dihydroxy-6-methylphthalide). Atranol exhibited the worst binding energy (see Table 4) and this is reflected in its experimental IC_50_ value.

In terms of molecular interactions among each compound and the residues of the catalytic site, all derivatives performed mainly hydrogen bond interactions, π–π interactions, T-shaped interactions, and hydrophobic interactions.

**Figure 4:**
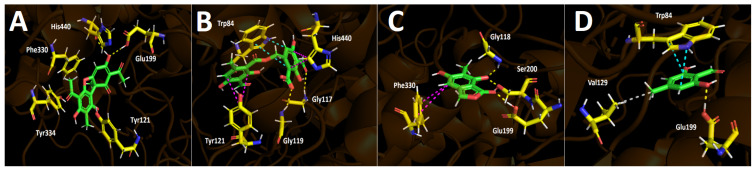
Predicted binding mode and predicted intermolecular interactions of the selected major compounds obtained from *H. lugubris* extract and the residues of the *Torpedo Californica* acetylcholinesterase (*Tc*AChE) catalytic site. Yellow dotted lines indicate hydrogen bond interactions, cyan dotted lines represent π–π interactions, magenta dotted lines represent T-shaped interactions, and grey dotted lines represent hydrophobic interactions. (**A**) Usnic acid in the catalytic site; (**B**) barbatolic acid in the catalytic site; (**C**) 5,7-dihidroxy-6-methylphthalide in in the catalytic site; (**D**) atranol in the catalytic site.

Usnic acid performs two hydrogen bond interactions with His440 and Glu199 through one of its phenolic hydroxyl groups (Figure 4A). Although this derivative just performs the two hydrogen bond interactions mentioned above, it carries out plenty of hydrophobic interactions with other catalytic amino acids, such as Trp84, Tyr 121, and Phe330, which allows its stabilization within the catalytic pocket, explaining its good potency profile. Barbatolic acid also showed two hydrogen bond interactions with Gly117 and Gly119, a π–π interaction between one of its benzene rings and Trp84, plus two T-shaped interactions with Tyr121 and His440 (Figure 4B). Even though this derivative performs more different interactions, its binding energy is less favorable, probably due to showing just a few hydrophobic interactions in the catalytic pocket compared to usnic acid. The compound 7-dihydroxy-6-methylphthalide shows three hydrogen bond interactions with Gly188 and Ser220 through one of the hydroxyl groups of its structure, and a third one between Glu199 and the carbonyl function that it possesses. Moreover, this compound also shows a T-shaped interaction with Phe330 which contributes to its fitting into the enzyme pocket. Notwithstanding, the same feature as in barbatolic acid can be seen, and not many hydrophobic interactions are executed by this compound (Figure 4C).

Finally, atranol showing the poorest binding energy and the highest IC_50_, exhibiting one hydrogen bond interaction with Glu199, one π–π interaction with Trp84, and just one hydrophobic interaction through its methyl group and the isopropyl function of valine amino acid (Figure 3D).

Finally, it could be suggested that the presence or absence of hydrophobic interactions are the main cause for the stabilization of derivatives within the acetylcholinesterase catalytic site.

#### 2.4.2. Butyrylcholinesterase (hBuChE) Docking Results

Binding energies from docking assays over butyrylcholinesterase (hBuChE) of usnic acid, barbatolic acid, 7-dihydroxy-6-methylphthalide, and atranol are in a close order of magnitude related to galantamine. As a matter of fact, the usnic acid derivative showed a slightly better energy compared to galantamine, but a large difference was not observed, suggesting the reason for the closer IC_50_ values between galantamine and usnic acid. Barbatolic acid represents an atypical case, since its binding energy value of −8.165 kcal/mol is better than the −7.125 kcal/mol shown by galantamine. The latter is not in agreement with the potency data obtained from the inhibitory assays over butyrylcholinesterase, the main reason probably being the largest number of functional chemical groups that this compound possesses compared to the others, which would increase its ability to perform and show more interactions in a docking assay. Therefore, this derivative must be considered as an outlier. On the other hand, 7-dihydroxy-6-methylphthalide and atranol exhibited worse energy profiles than galantamine, suggesting that usnic acid is the best butyrylcholinesterase inhibitor.

The main intermolecular interactions in the butyrylcholinesterase catalytic site and the different derivatives are: hydrogen bond interactions, π–π interactions, T-shaped interactions, π–cation interactions, salt bridges, and hydrophobic interactions.

**Figure 5:**
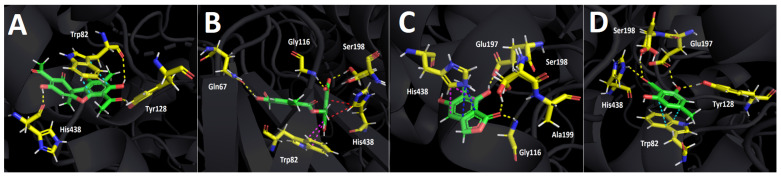
Predicted binding mode and predicted intermolecular interactions of the selected major compounds obtained from *H. lugubris* extract and the residues of human butyrylcholinesterase (*h*BuChE) catalytic site. Yellow dotted lines indicate hydrogen bond interactions, cyan dotted lines represent π–π interactions, magenta dotted lines represent T-shaped interactions, blue dotted lines represent π–cation interactions, and red dotted lines represent salt bridges. (**A**) Usnic acid in the catalytic site; (**B**) barbatolic acid in the catalytic site; (**C**) 5,7-dihidroxy-6-methylphthalide in in the catalytic site; (**D**) atranol in the catalytic site.

Usnic acid binding descriptors over butyrylcholinesterase, as in acetylcholinesterase docking results, showed two hydrogen bond interactions with the amino acids Tyr128 and His438, and a π–π interaction can also be seen between Trp82 and the benzene framework of this compound (Figure 5A). Docking experiments showed that barbatolic acid had three hydrogen bond interactions through its different oxygenated functional groups and Gln67, Gly116, and Ser198 residues. In the same manner, it also exhibited one T-shaped interaction and a salt bridge; nonetheless, as was mentioned above, these binding descriptors must be considered carefully to obtain conclusions (Figure 5B).

The compound 7-Dihydroxy-6-methylphthalide, which demonstrates a similar IC_50_ value compared to barbatolic acid over butyrylcholinesterase inhibitory experiments (19.47 ± 0.10 ppm and 23.95 ± 0.06, respectively), showed three hydrogen bond interactions with Gly116, Glu197, and Ser198, as well as T-shaped and π–cation interactions. Notwithstanding, no salt bridge was found for this derivative, opening the possibility that the salt bridge could improve the energy profile of barbatolic acid (Figure 5C).

Once again, atranol showed the worst binding energy (−6.343 kcal/mol); hence, it is normal that its IC_50_ value was 36.43 ± 0.08 ppm. Since this derivative corresponds to a simple and small hydroxylated aldehyde, it probably possesses lower abilities to fit and stabilize inside the catalytic pocket. Atranol showed four hydrogen bond interactions with Tyr198, Glu197, Ser198, and His438 through one of its hydroxyl groups and by the aldehyde that it contains. A π–π interaction with Trp82 can also be contemplated (Figure 5D). Furthermore, only two hydrophobic interactions for this compound with Trp82 and Leu125 can be seen.

#### 2.4.3. Tyrosinase (Tyr) Docking Results

Binding energies from docking assays over tyrosinase of usnic acid, barbatolic acid, 7-dihydroxy-6-methylphthalide, and atranol showed, in general terms, similar or slightly worse values than the known inhibitor kojic acid. The latter would explain the obtained potency values (IC_50_) over the enzymatic inhibitory activity of compounds (Table 4). As in butyrylcholinesterase docking assays, barbatolic acid exhibited a higher binding energy than kojic acid, specifically −6.490 kcal/mol. Again, this result must be considered as an outlier as it does not agree with the enzymatic inhibition evidence from Table 2. The better energy profile of barbatolic acid probably comes from the functional chemical groups that it bears, as was suggested before.

On the other hand, usnic acid exhibits an IC_50_ value of 132.23 ± 0.12 ppm, showing less ability to inhibit the tyrosinase compared to its ability to inhibit the cholinesterases. This feature agrees with the binding energy obtained in docking assays for this derivative (−5.744 kcal/mol). Even if its energy is better than those shown by 7-dihydroxy-6-methylphthalide and atranol, all the energy values are in a close range, so no substantive differences can be seen for this docking descriptor.

**Figure 6:**
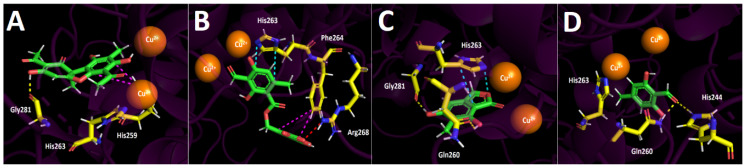
Predicted binding mode and predicted intermolecular interactions of the selected major compounds obtained from *H. lugubris* extract and the residues of the *Agaricus bisporus* mushroom tyrosinase catalytic site. Yellow dotted lines indicate hydrogen bond interactions, cyan dotted lines represent π–π interactions, magenta dotted lines represent T-shaped interactions, and red dotted lines represent salt bridges. (**A**) Usnic acid in the catalytic site; (**B**) barbatolic acid in the catalytic site; (**C**) 5,7-dihidroxy-6-methylphthalide in in the catalytic site; (**D**) atranol in the catalytic site.

In terms of molecular interactions among each compound and the residues of the tyrosinase catalytic site, docking experiments displayed the following results. Usnic acid showed one hydrogen bond interaction with Gly281 and a T-shaped interaction between its benzene framework and the imidazole ring of His259 (Figure 6A). Barbatolic acid in the tyrosinase catalytic site showed a π–π interaction between its dihydroxymethylbenzaldehyde core and His263, a T-shaped interaction between its formyl-dihydroxybenzoic acid framework and Phe264, but also a salt bridge with Arg268, which is protonated, and the carboxylate function that barbatolic acid bears (Figure 6B).

Furthermore, 7-Dihydroxy-6-methylphthalide and atranol possess similar binding energies. In the same sense, the IC_50_ values of these two derivatives were in the same range (12.13 ± 0.15 ppm and 7.25 ± 0.18 ppm, respectively). Within the tyrosinase active site, both compounds showed two hydrogen bond interactions, but 7-dihydroxy-6-methylphthalide also showed an extra π–π interaction that atranol did not execute. The two hydrogen bond interactions shown by 7-dihydroxy-6-methylphthalide mentioned above were with Gln260 and Gly281, while the π–π interaction was through His263 (Figure 6C). On the other hand, the two hydrogen bond interactions exhibited by atranol were with the residues of Gln269 and His244 (Figure 6D).

**Figure 7:**
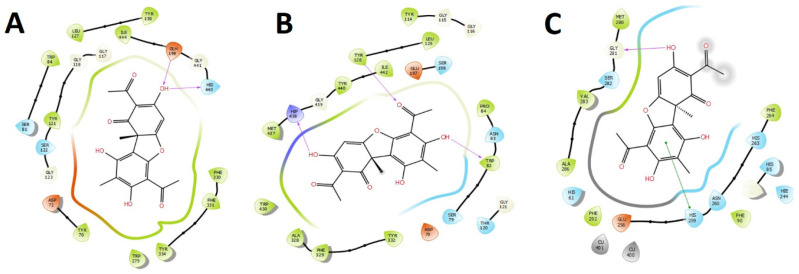
Two-dimensional diagram of (**A**) usnic acid in the acetylcholinesterase (*Tc*AChE) catalytic site, (**B**) usnic acid in the butyrylcholinesterase (*h*BuChE) catalytic site, and (**C**) usnic acid in the tyrosinase catalytic site. Magenta arrows represent hydrogen bond interactions; green lines represent T-shaped interaction. Orange amino acids indicate negative charged residues, magenta amino acids indicate positive charged residues, green amino acids indicate hydrophobic residues, and light blue amino acids indicate polar residues.

Finally, Figure 7 summarizes the binding mode and the intermolecular interactions of usnic acid, which, according to our docking results and the enzymatic inhibitory experiments, was the most active compound inhibiting the acetylcholinesterase and butyrylcholinesterase. Figure 6 also shows the binding mode and the intermolecular interactions of usnic acid in the tyrosinase catalytic site since it turned out to be the least active in this enzyme.

## 3. Materials and Methods

### 3.1. Chemicals

Ultra-pure water (<5 µg/L TOC) was obtained from a purification system (Arium 126 61316-RO), plus an Arium 611 UV unit (Sartorius, Goettingen, Germany). HPLC grade methanol and formic acid (for mass spectrometry) were obtained from J. T. Baker (Phillipsburg, NJ, USA). Commercial Folin–Ciocalteu reagent, 2,2-diphenyl-1-picrylhydrazyl (DPPH), ferric chloride hexahydrate, 2,4,6-tris(2-pyridyl)-s-triazine, Trolox, quercetin, gallic acid, dimethyl sulfoxide (DMSO), Amberlite^®^ resin (XAD-4), tyrosinase (EC1.14.18.1), acetylcholinesterase (AChE, EC 3.1.1.7), butyrylcholinesterase (BChE, EC 3.1.1.8), phosphate buffer, L-DOPA, kojic acid, trichloroacetic acid (Merck, Darmstadt, Germany), fetal calf serum (FCS, Gibco, Grand Island, NY, USA), L-glutamine (Merck, Darmstadt, Germany), sodium carbonate, ferrous sulfate, sodium persulfate, sodium acetate, sodium sulfate anhydrous, and absolute ethanol were obtained from Sigma (Sigma, St. Louis, MO, USA) and HPLC standards (usnic acid, barbatolic acid purity 98% by HPLC) were obtained from Extrasynthèse (Genay, France), Phytolab (Vestenbergsgreuth, Germany), Biopurify (Chengdu, China), or BOCSY (Shirley, NY, USA). The NMR experiments (^1^H: 300.12 MHz; ^13^C: 100.25 MHz) were performed using a Bruker Avance 300 with CD_3_OD or deuterated MeOD as solvent and TMS as internal standard (Pontificia Universidad Católica de Chile).

### 3.2. Plant Material

*H. lugubris* was collected by hand by M.J.S and A.T-B. from Ardley Island, King George, Antarctica, in February 2021 (Chilean Antarctic expedition, ECA 57th). The sample was authenticated by the botanist Alfredo Torres-Benitez. A voucher specimen (voucher number H.l-01152021) was recorded and placed in the Laboratory of Natural Products of the Universidad Austral de Chile (Valdivia, Chile).

### 3.3. Extraction and Isolation Procedure

#### 3.3.1. Extraction

Dried and ground *H. lugubris* (500 g) was extracted with ethanol in an ultrasonic water bath (UC-60A Biobase, Jinan, Shandong, China) at room temperature for 30 min in the dark (1 L fresh absolute ethanol each time, three times). The extract was filtered and evaporated under reduced pressure at 36 °C to obtain the ethanolic extract (28 g).

#### 3.3.2. Isolation

The extract (22 g) was submitted to open column chromatography (300 g of Kieselgel 60 H), and chromatographed with hexane: ethyl acetate of increased polarity to gather fractions (A-D) which were collected together according to TLC analysis (silica gel plates F254, and hexane acetate 7:3 *v*:*v*, revealed with vanillin 1 percent in EtOH: sulfuric acid 85:5% *v*:*v*). From fraction B (3 g) by medium pressure column chromatography (Kieselgel 60 G, and hexane acetate 80:20 *v*:*v*), 335 mg of usnic acid **1** and 33 mg of barbatolic acid **2** were obtained, and from fraction C by chromatography (Kieselgel 60 G, and isocratic hexane acetate 90:10 *v*:*v*), 27 mg of 5,7-dihydroxy-6-methylphthalide **3** and 221 mg of atranol **4** were obtained (Figure 2). Usnic acid: (300 MHz, CDCl_3_): 1.76 (3H, s, Me-13), 2.11 (3H, s, Me-16), 2.66 (3H, s, Me-15), 2.77 (3H, s, Me-18), 5.95 (lH, s, H-4), 11.30 (lH, s, HO-10), 14.36 (lH, s, HO-8), 18.70 (lH, s, HO-3) [55]; barbatolic acid: ^1^H-NMR (300 MHz, CDCl_3_-DMSO-d6): 2.44 (3H, s, Me-9), 5.28 (2H, s, -CH_2_-9’), 6.36 (lH, s, H-5), 6.48 (2H, s, H-l’, H-5’), 10.34 (lH, s-CHO-8); 5,7-dihydroxy-6-methylphthalide: ^1^H-NMR (300 MHz, CDCl_3_): 2.22 (3H, s, Me), 6.45 (lH, s, arom.-H), 5.35 (CH_2_-), 9.50 (lH, s, -OH), 12.06 (lH, s, -OH) [56]; atranol: ^1^H-NMR (300 MHz, CDCl_3_): 2.31 (3H, s, Me), 6.32 (lH, s, arom. -H), 6.40 (lH, s, -OH), 10.20 (lH, s, CHO), 11.08 (lH, s, -OH) [57]. The purity of the isolated compounds (>95%) was determined based on HPLC-PDA analysis.

### 3.4. UHPLC–DAD–MS Instrument

A UHPLC-high-resolution MS machine (Thermo Dionex Ultimate 3000 system with PDA detector controlled by Chromeleon 7.2 software hyphenated with a Thermo Q-Exactive MS focus, (Thermo Fisher Scientific, Bremen Germany was used to determine the compounds in the extract. For the analysis, 5 mg of the ethanolic extract was dissolved in 2 mL of methanol, filtered through a 200 µm polytetrafluoroethylene (PTFE) filter, and 10 µL was injected in the instrument [58].

### 3.5. LC Parameters and MS Parameters

A UHPLC Acclaim C18 column (150 × 4.6 mm ID, 2.5 µm; Thermo Fisher Scientific, Bremen, Germany) operated at 25 °C was employed. Four detection wavelengths were at 280, 254, 330, and 354 nm, and diode array detectors were at 200–800 nm. Mobile phases were 1% formic acid in water (A) and acetonitrile (B). The gradient program started at 5% B for 5 min, then went up to 30% B for 10 min, then 30% B for 15 min, then up to 70% B for 5 min, then maintained 70% B for 10 min, and came back to initial conditions in 12 min for column equilibration. The flow rate was 1.00 mL/min, and the injection volume was 10 µL. Standards and the extract dissolved in ethanol were kept at 10 °C during storage. The HESI II and Orbitrap spectrometer parameters were optimized as previously reported [54]. The chromatographic system was coupled to a spectrometer with a source II heated electro-nebulization HESI II probe. The mass calibration for Orbitrap was performed every day, with operating mass equal to 5 ppm. A mixture of buspirone hydrochloride, taurocholic acid sodium salt, and sodium dodecil sulfate (Sigma-Aldrich, Darmstadt, Germany), plus 1621 mass units fluorinated phosphazine solution (Ultramark 1621Alpha Aezar, Stevensville, MI, USA), was used as the standard mixture for calibration. Xcalibur 2.3 and Trace Finder 3.2 (Thermo Fisher Scientific, Bremen, Germany) were used for UHPLC mass spectrometer control and data processing, respectively.

### 3.6. HPLC Quantitative Analysis

Quantification of some major compounds in *H. lugubris* ethanol extract (usnic acid, barbatolic acid, and atranol) was performed (Table 1). The content of compounds in the extracts was established with reference to the calibration curves of the compounds performed at 280 nm and 15 min for column equilibration. A curve was performed with each of the individual standards (LOD: 2.35, LOQ: 8.91 mg/L for usnic acid, quantified at 280 nm, standard recovery of usnic acid: 99.12%; LOD: 2.53, LOQ: 9.21 mg/L for barbatolic acid, standard recovery of this acid: 98.22%). Measurements were performed in triplicate at each concentration level (5, 10, 20, 40, 60, 60, 80, 100 mg/mL) and the equation of the curve and the coefficient of determination were obtained (for usnic acid, y = 0.0056x + 0.1327, R^2^ = 0.9991; for barbatolic acid, y = 0.011x + 0.0586, R^2^ = 0.9993; and for atranol, y = 0.2682x + 1.6170, R^2^ = 0.9996) with Chromeleon 7.2 software. The results were expressed as the mean ± standard deviation (SD).

### 3.7. Total Phenolic (TP) Content

The total phenolic (TP) content of *H. lugubris* was measured using the Folin–Ciocalteu by using a Synergy HTX microplate reader (Biotek, Winoosky, VT, USA) [47,59]. Results (TP) are expressed as mg gallic acid equivalent per g of dry lichen. The experiments were performed in triplicate and the values are reported as the mean ± SD.

### 3.8. Antioxidant Activity

#### 3.8.1. DPPH Scavenging Activity

The potential bleaching of the radical DPPH, using a Synergy HTX microplate reader, was determined by mixing 50 µL of extract or standard with 150 µL of DPPH solution; the determinations were made in triplicate by monitoring the bleaching of DPPH at 515 nm after 30 min of reaction [54,58]. The results are expressed as IC_50_ in µg of standard or extract per mL. The data are reported as the mean ± SD.

#### 3.8.2. Ferric-Reducing Antioxidant Power Assay (FRAP)

The FRAP test was performed as described previously [20]. A standard curve of the antioxidant Trolox was plotted, then the measurement was performed with 290 µL of extract and its absorbance was measured at 593 nm after 5 min. The results were expressed in mg of Trolox equivalent per g of dry lichen. The experiments were performed in triplicate and the values are expressed as the mean ± SD.

#### 3.8.3. Oxygen Radical Absorbance Capacity (ORAC) Assay

The ORAC assay was performed as previously described [60]. Quantification was performed using a standard curve of the antioxidant Trolox, then the measurement was performed using a solution of the Trolox, blank, and 40 µL of extract in different wells of the microplate reader. The results were obtained by constructing the equations (Trolox/samples vs. fluorescence decay curves) and expressed in μmol Trolox equivalents per grams of dry lichen. The tests were performed in triplicate and the results are reported as the mean ± SD.

### 3.9. Determination of Cholinesterase Inhibition

Inhibition of AChE activity was determined according to the Ellman method, as previously reported [54]. The samples (2 mg/mL) were prepared by mixing tris-HCl buffer (50 mM, pH 8.0), enzyme solution (AChE or BChE), and 5-dithio-bis (2-nitrobenzoic acid) (DTNB) at pH 8.0. The reaction was started by adding acetyl-thiocholine iodide (ATCI) or butyryl-thiocholine chloride (BTCl). The absorbance of the extract was measured at 405 nm for 30 min at 37 °C. Three experiments were performed in triplicate in each test and the values are reported as the mean ± SD. The results were expressed as IC_50_ (µg/mL, the final concentration of the plate ranged from 0.05 to 25 μg/mL). Galantamine was used as a positive control.

### 3.10. Tyrosinase Inhibition Assay

Tyrosinase activity was assessed by the employment of the dopachrome method [61]. *H. lugubris* extract or pure compound (20 µL in EtOH, at 1 mg/mL) with 30 µL of pH 6.8 phosphate buffer (0.067 M, pH 6.8), 40 µL of the enzyme tyrosinase (100 U/mL), and 40 µL L-DOPA (2.5 mM) as substrate were added in each well. The reaction was incubated for 15 min at 24 °C and the absorbance obtained at 492 nm. The results were expressed as IC_50_ (µg/mL, the final concentration of the plate ranged from 31.25 to 250 μg/mL), the experiments were performed three times, and the values are reported as the mean ± SD. The positive control compound was kojic acid.

### 3.11. Docking Simulations

Docking simulations were carried out for selected major compounds shown in Figure 2 obtained from *H. lugubris* extract. First, full optimization was conducted using the DFT method of the partial charges and geometries of each compound with the standard basis set B3LYP-6-311G+ (d p) [62,63] in the Gaussian 09W software [64]. Then, minimizations of their energies and protonation or deprotonation (if applicable) were carried out using the LigPrep tool in Maestro Schrödinger suite v.11.8 (Schrödinger, LLC) [65]. Crystallographic enzyme structures of Torpedo Californica acetylcholinesterase (TcAChE; PDBID: 1DX6 code [66]), human butyrylcholinesterase (hBuChE; PDBID: 4BDS code [67]), and the *Agaricus bisporus* mushroom tyrosinase (tyrosinase; PDBID: 2Y9X code) [68] were obtained from the Protein Data Bank RCSB PDB [69]. Enzyme optimizations were performed using the Protein Preparation Wizard available in the Maestro software, and then water molecules and ligands of the crystallographic protein active sites were removed. In the same way, all polar hydrogen atoms at pH = 7.4 were added. Appropriate ionization states for basic amino acid residues and acid residues were considered. The OPLS3e force field was employed to minimize protein energy. The enclosing box size was optimized to a cube with sides of 26 Å length. The centroid of selected residues was chosen based on the assumed catalytic site of each enzyme, considering their known catalytic amino acids: Ser200 for acetylcholinesterase (TcAChE) [70,71], Ser198 for butyrylcholinesterase (hBuChE) [72,73], and His263 for tyrosinase [74,75]. The Glide Induced Fit Docking protocol was set up for the final couplings [76]. Compounds were punctuated by the Glide scoring function in the extra-precision mode (Glide XP; Schrödinger, LLC) [77] and were filtered based on the best scores and best RMS values (less than 1 unit as a cutting criterion), to obtain the prospective intermolecular interactions between the compounds and the enzymes, and the binding mode and docking descriptors. The different complexes were envisioned in the visual molecular dynamics program (VMD) and Pymol [78].

### 3.12. Statistical Analysis

The results obtained from these experiments were indicated as mean ± standard error of mean. Statistical analysis of the data was done using analysis of variance (one-way ANOVA). In addition, the determination of the reactivity (EC_50_ or IC_50_) was obtained using sigmoidal nonlinear regression via Graph Pad Prism software, version 5.0. (GraphPad Software, Inc., La Jolla, CA, USA). Statistical significance was set at *p* = 0.05.

## 4. Conclusions

The antioxidant and enzyme inhibition potential (against tyrosinase and cholinesterase) and the phenolic fingerprinting of *H. lugubris* from Antarctica and four major isolated compounds were investigated for the first time. High-resolution mass spectrometry (UHPLC-PDA-Orbitrap-MS) was used to detect 28 metabolites. To the best of our knowledge, this is the first investigation on the inhibitory activity (against cholinesterase and tyrosinase) of this Antarctic lichen and its main components. The results of the enzymatic inhibitory activity showed a moderate inhibition of the extracts and good inhibition of phenolic isolated constituents. These results generally illustrate the potential of this phenolic enriched lichen to produce an extract with both nutritional and health-promoting properties that can be used in neurodegenerative or related chronic non-transmittable diseases. Bioassay-guided fractionation and further isolation of minor compounds are needed to determine other bioactive compounds responsible for these activities from this Antarctic species.

## Figures and Tables

**Table 1:** Identification of phenolic compounds by HESI orbitrap HR-MS of *Himantormia lugubris* ethanolic extract.

Peak	Retention Time (min.)	Tentative Identification	[M-H]^−^	Theoretical Mass (*m*/*z*)	Measured Mass (*m*/*z*)	Accuracy (ppm)	Metabolite Type	MS Ions (ppm)
1	1.34	Mannitol	C_6_H_13_O_6_	181.0712	181.0705	3.9	CH	151.0598
2	1.78	Citric acid	C_6_H_7_O_7_	191.0192	191.0184	4.2	CH	111.0074
3	11.43	Atranol *	C_8_H_7_O_3_	151.0395	151.0387	5.3	A	135.0438; 123.0438; 107.0488
4	12.91	5,7-Dihydroxy-4-methylphthalide	C_9_H_7_O_4_	179.0344	179.0336	4.5	A	107.0488; 135.0437; 151.0386
5	13.18	Haematommic acid (3-formyl-2,4-dihydroxy-6-methylbenzoic acid)	C_9_H_7_O_5_	195.0293	195.0286	3.6	A	179.0335; 151.0387; 123. 0438; 149.0230
6	14.93	5,7-Dihydroxy-6-methylphthalide *	C_9_H_7_O_4_	179.0344	179.0337	3.9	A	135.0438; 107.0488
7	16.28	9,10,12,13-Tetrahydroxyheneicosanoic acid	C_21_H_41_O_6_	389.2903	389.2892	2.8	L	371.2784
8	17.35	9,10,12,13,14-Pentahydroxytetracosanoic acid	C_24_H_47_O_7_	447.3322	447.3306	3.6	L	389.2891; 429.3199; 361.2581
9	18.71	Evernic acid isomer (3-hydroxy-4-(2-hydroxy-4-methoxy-6-methylbenzoyl)oxy-6-methylbenzoic acid)	C_17_H_15_O_7_	331.0818	331.0809	2.7	d	135.0438; 123.0439; 181.0494 151.0386; 167.0336; 313.0703
10	19.75	Methyl orsellinate	C_9_H_9_O_4_	181.0501	181.0494	3.9	A	151.0387; 123,0439; 135.0438
11	19.83	9,10,12,13-Tetrahydroxydocosanoic acid	C_22_H_43_O_6_	403.3060	403.3047	3.2	L	385.2939; 215.1273
12	20.16	Evernic acid II isomer (3-hydroxy-4-(3-hydroxy-4-methoxy-6-methylbenzoyl)oxy-6-methylbenzoic acid)	C_17_H_15_O_7_	331.0818	331.0809	2.7	d	195.0284; 151.0386; 123.0438; 135.0436; 167.0336
13	20.25	Pentahydroxyhexacosanoic acid	C_26_H_51_O_7_	475.3635	475.3618	3.6	L	-
14	20.33	9,10,12,13-Tetrahidroxytricosanoic acid	C_23_H_45_O_6_	417.3236	417.3204	7.7	L	399.3095
15	20.42	Barbatolic acid *	C_18_H_13_O_10_	389.05080	389.05086	2.5	A	211.0246, 195.02122
16	20.49	Isomer haematommic acid (3-formyl-2,5-dihydroxy-6-methylbenzoic acid)	C_9_H_7_O_5_	195.0293	195.0285	4.1	A	179.0335; 151.0387; 123.0438
17	20.81	2,4-Diformyl-3,5-dihydroxytoluene o 2,6-Diformyl-3,5-dihydroxytoluene	C_9_H_7_O_4_	179.0344	179.0338	3.4	A	151.0386; 107.0488; 135.0437
18	21.74	Evernic acid (2-hydroxy-4-(2-hydroxy-4-methoxy-6-methylbenzoyl)oxy-6-methylbenzoic acid)	C_17_H_15_O_7_	331.0818	331.0808	3.0	d	167.0334; 151.0386; 135.0437 313.0703; 123.0439; 181.0494
19	21.85	9,10,12,13,14,15-Hexahydroxyheptacosenoic acid	C_27_H_51_O_8_	503.3584	503.3564	4.0	L	475.3615; 443.3355
20	22.30	Methyl 9,10,11,12,13-pentahydroxy-14-oxoheptacosanoate	C_28_H_53_O_8_	517.3740	517.3719	4.1	L	457.3510; 439.3404
21	23.08	Lichesterinic acid o Protolichesterinic acid	C_19_H_31_O_4_	323.2222	323.2213	2.8	L	279.2315; 267.2314
22	23.49	Tetrahydroxydioxoheneicosanoic acid	C_21_H_37_O_8_	417.2494	417.2496	0.9	L	399.3091
23	23.85	Tetrahydroxydocosanoic acid	C_22_H_43_O_6_	403.3065	403.3067	0.6	L	-
24	24.30	Sphaerophorin	C_23_H_27_O_7_	415.1757	415.1744	3.1	d	233.1166; 207.1376; 251.1275
25	24.67	Pseudoplacodiolic acid or Placodiolic acid	C_19_H_19_O_8_	375.1080	375.1070	2.7	DBF	343.0807; 259.0598; 231.0648
26	25.63	Isomer barbatic acid (3-hydroxy-4-(2-hydroxy-4-methoxy-3,6-dimethylbenzoyl)oxy-3,6-dimethylbenzoic acid)	C_19_H_19_O_7_	359.1131	359.1121	2.8	d	181.0493; 163.0387; 137.0594
27	25.78	Barbatic acid (2-hydroxy-4-(2-hydroxy-4-methoxy-3,6-dimethylbenzoyl)oxy-3,6-dimethylbenzoic acid)	C_19_H_19_O_7_	359.1131	359.1120	3.1	d	181.0493; 163.0387; 137.0594
28	26.16	Usnic acid *	C_18_H_15_O_7_	343.0818	343.0808	2.9	DBF	259.0598; 231.0647; 328.0570

* Identified by spiking experiments with an authentic standard compound. CH = carbohydrates; A = aromatic; L = lipid; D = depsidone; d = depside; DE = diphenyl ether; DBF = dibenzofuran; C = chromone.

**Table 2:** Quantitation of main compounds in of *H. lugubris* ethanolic extract (mg/g dried lichen).

	Usnic Acid *	Barbatolic Acid *	Atranol *	5,7-Dihydroxy-6-methylphthalide *
*H. lugubris* lichen	8.921 ± 0.372	85.833 ± 0.325	32.345 ± 0.071	49.374 ± 0.095

* (mg/g dried lichen).

**Table 3:** Total phenolic content (TPC), antioxidant activity (FRAP; ORAC, DPPH) and enzymatic inhibitory activity of *H. lugubris*.

Assay	TPC ^a^	FRAP ^b^	ORAC ^b^	DPPH ^c^	AChE ^d^	BChE ^d^	Tyr ^d^
*H. lugubris* Ethanol extract	47.4 ± 0.05	27.8 ± 0.0	32.7 ± 0.70	75.3 ± 0.02	12.38 ± 0.09 ^b^	31.54 ± 0.20	22.32 ± 0.21
Usnic acid		22.4 ± 0.00	122.73 ± 1.0	55.25 ± 0.04	2.21± 0.03	4.36 ± 0.03	132.23 ± 0.12
Barbatolic acid	-	28.10 ± 0.0 ^a^	101.11 ± 0.71	62.55 ± 0.01	17.42 ± 0.03	23.95 ± 0.02	35.23 ± 0.11
Atranol	-	29.32 ± 0.0 ^a^	176.28 ± 0.84	21.04 ± 0.02	28.82 ± 0.10	36.43 ± 0.08	7.25 ± 0.18
5,7-dihydroxy-6-methylphthalide	-	36.91 ± 0.0	373.65 ± 1.05	12.52 ± 0.02	12.71 ± 0.12 ^b^	19.47 ± 0.10	12.13 ± 0.15
Gallic acid	-	45.5 ± 0.00	-	2.24 ± 0.04	-	-	
Galantamine	-	-	-		0.27 ± 0.03	3.82 ± 0.02	-
Kojic acid	-	-	-	-	-	-	0.76 ± 0.05

Each value represents the means ± SD of three replicates, n = 3, while the same letters in the same column indicate no significative difference using the Tukey test at 0.05 level of significance (*p* < 0.05). ^a^ Total phenolic content (TPC) expressed as mg GAE equivalent/g dry weight. ^b^ Expressed as μmol Trolox/g lichen. ^c^ Antiradical DPPH activities are expressed as μg/mL. ^d^ Expressed as IC_50_ in µg/mL. FRAP, ferric reducing/antioxidant power; ORAC, oxygen radical absorbance capacity; DPPH, 2,2-diphenyl-1-picryl-hydrazyl-hydrate; AChE, acetylcholinesterase; BChE, butyrylcholinesterase; Tyr, tyrosinase.

**Table 4:** Binding energies obtained from docking experiments of selected major compounds in *H. lugubris* as well as the known inhibitor binding energy galantamine over acetylcholinesterase (TcAChE), butyrylcholinesterase (*h*BuChE), and tyrosinase.

Compound	Binding Energy (kcal/mol) Acetylcholinesterase (*Tc*AChE)	Binding Energy (kcal/mol) Butyrylcholinesterase (*h*BuChE)	Binding Energy (kcal/mol) Tyrosinase
Usnic acid	−10.779	−8.844	−5.744
Barbatolic acid	−8.027	−8.165	−6.490
7-dihydroxy-6-methylphthalide	−7.913	−6.855	−4.639
Atranol	−6.197	−6.343	−4.964
Galantamine	−12.989	−7.125	-
Kojic acid	-	-	−6.050

## Data Availability

The raw HPLC-MS and NMR data presented in this study are available on request from the corresponding author. The data are not publicly available due to large or heavy raw files.
